# Short- and long-term impact of aseptic bathing strategies on the skin microbiome in ICU patients

**DOI:** 10.1007/s00430-025-00843-1

**Published:** 2025-07-22

**Authors:** Tilman E. Klassert, Cristina Zubiria-Barrera, Luisa A. Denkel, Mercedes Lopez, Robert Neubert, Amelya Keles Slevogt, Frank Bloos, P. Christian Schulze, Jörg Epstude, Petra Gastmeier, Christine Geffers, Hortense Slevogt

**Affiliations:** 1 Department of Respiratory Medicine and Infectious Diseases, Hannover Medical School, German Center for Lung Research (DZL), BREATH, Hannover, Germany; 2 Dynamics of Respiratory Infections Group, Helmholtz Centre for Infection Research– HZI, Inhoffenstraße 7, 38124 Braunschweig, Germany; 3 Institute for Hygiene and Environmental Medicine, Charité - Universitätsmedizin Berlin, Berlin, Germany; 4 Genomics and Health Group, University of La Laguna, San Cristóbal de La Laguna, Spain; 5 Department of Neurology and Experimental Neurology, Charité - Universitätsmedizin Berlin, Berlin, Germany; 6 Department of Anesthesiology and Intensive Care Medicine, Jena University Hospital, Jena, Germany; 7 Department of Internal Medicine I, Cardiology, Angiology, Intensive Medical Care, University Hospital Jena, Jena, Germany; 8 Department of Hospital Hygiene, Thuringia Clinic “Georgius Agricola”, Saalfeld/Saale, Germany; 9 Cluster of Excellence RESIST (EXC 2155), Hannover Medical School, Carl-Neuberg-Straße 1, 30625 Hannover, Germany

**Keywords:** Bathing, Skin microbiome, ICU, Critical illness

## Abstract

Bathing strategies with antiseptic agents, such as Chlorhexidine and Octenidine, have been widely adopted to mitigate infection risks in intensive care units (ICU). However, concerns exist regarding their long-term effects on skin microbiome structures and potential unintended consequences, including antibiotic cross-resistance. This longitudinal study characterized the compositional changes of the skin microbiome of ICU patients upon these two antiseptic bathing strategies when compared to standard water and soap bathing. Samples were collected in a three-armed cluster randomized decolonization trial (registration number DRKS00010475). Skin swabs from 5 different sites and three time points were analyzed by culture-based methods, 16S rRNA-gene amplicon sequencing and multiplex Taq-Man assays for detection of antimicrobial resistance genes (ARG). Our results show that Chlorhexidine bathing led to a sustained reduction of the bacterial biomass on different skin sites, as measured by both molecular and culture-based methods. Thereby, the microbial structures remained largely unaltered both in their diversity and their taxonomic composition. However, the loss of microbiome site-specificity observed on the skin of ICU patients remained unchanged independently from the bathing strategy applied and persisted even after discharge. None of the antiseptic bathing strategies led to an increase or accumulation of antibiotic-resistance determinants on any of the skin sites investigated in this study. Thus, this study suggests that daily patient bathing with 2% Chlorhexidine impregnated cloths or 0.08% Octenidine wash mitts does not impact skin microbiome structures and antibiotic resistance gene accumulation in ICU patients when compared to non-antiseptic water and soap bathing routine.

**Supplementary Information:**

The online version contains supplementary material available at 10.1007/s00430-025-00843-1.

## Introduction

The skin of patients represents a major reservoir for pathogens associated with nosocomial infections, and has been proposed as target for different interventions to reduce bacterial burden and risk of infection [1, 2]. Patients in the intensive care units (ICU) are particularly susceptible to pathogenic colonization of the skin and subsequent infection is often triggered by recurrent contact with invasive devices and compromised host defenses due to antibiotic exposure and skin barrier damage [3, 4]. Moreover, our group could recently demonstrate that at admission, the skin microbiota of ICU patients is already in dysbiosis, characterized by a loss of microbiome site-specificity and an overrepresentation of gut bacteria [5]. This condition might further bolster the risk of infection in this susceptible cohort of patients, since the array of distinct microbial communities on the skin of healthy individuals has shown to play pivotal roles in the protection against pathogenic threats and the modulation of the immune system [6].

A growing empirical evidence suggests that bathing with broad-spectrum antiseptics such as Chlorhexidine and Octenidine can effectively reduce infection rates in the short term, underscoring their contribution to patient safety and mitigating the burden of nosocomial infections in ICU settings [1, 7–10]. However, there is also a growing evidence that these antiseptic strategies might have unintended consequences, such as increased tolerance to disinfectants or increase of antibiotic cross-resistances [11–13]. While susceptibility of bacterial isolates from clinical samples to Chlorhexidine and Octenidine did not change after daily bathing with these substances for one year [14], there is still a critical gap in understanding their long-term effects on skin microbiome structures [15]. Concerns persist regarding the potential for microbial dysbiosis and alterations to colonization resistance following prolonged exposure to antiseptic bathing regimens. Addressing this knowledge gap is essential for refining infection control practices and optimizing patient outcomes in critical care settings.

This longitudinal study was designed to answer the question whether daily patient bathing with 2% Chlorhexidine impregnated cloths or 0.08% Octenidine wash mitts might impact skin microbiome structures and antibiotic resistance gene accumulation in ICU patients when compared to non-antiseptic water and soap bathing routine. The research also aimed at elucidating the long-term impacts of these antiseptic regimens on microbial dynamics within the various hospital settings.

## Material and methods

### Study design

This longitudinal study was performed as part of the project Climate and pathogens–impact of decolonization (CLIP-ID, registration number DRKS00010475). The microbiome-analyses covered in this subproject were designed to characterize the differential effect of three different bathing strategies on the skin microbiome composition in ICU-patients. The study's workflow is detailed in the Suppl. Fig. 1, which illustrates the sequential steps and methodologies utilized throughout the research. This research was part of the ICU-KISS network [16], a hospital-based surveillance system in Germany that compiles data on nosocomial infections (NIs) from approximately 1,000 voluntarily participating intensive care units. Within the cluster-randomized decolonization trial, 72 ICUs were randomly assigned to daily patient bathing with Chlorhexidine, Octenidine or water and soap (as control). For our study, we selected one ICU per study arm, each employing a distinct bathing strategy. Hospital A was attributed to water and soap; hospital B utilized 2% Chlorhexidine-impregnated cloths (Stryker); and hospital C implemented 0.08% Octenidine (Octenisan/Schülke & Mayr) disposable wash mitts. Details on daily bathing protocols were explained elsewhere [7, 10, 14]. All ICUs, regardless of group allocation, were provided with group-specific standardized operating procedures and 3 min training videos to ensure consistency in bathing practices. Across all groups, daily bathing by trained nursing staff was recommended. Compliance with the bathing protocols was assessed through on-site visits to selected wards in each group and by monitoring the consumption of antiseptic products in the intervention groups. Wards consumed on the average 1.3 packages per patient day of Chlorhexidine-impregnated cloths (with six cloths per package) and 1.1 packages per patient day of Octenidine wash mitts (with 10 wash mitts per package). Patients with an anticipated ICU-stay of 5 days or more days were eligible for this study and asked for their consent to participate. Sampling was performed at three different time points: (i) within 12 h after admission (T1); (ii) during the ICU-stay right before the 5th bathing (T2); and (iii) 5 days after discharge of the patient (T3). Final sampling for patients discharged from the ICU was conducted on non-ICU wards within the same hospital. In total, twenty-six ICU-patients (age range 25–90 years; η = 62.5 years) were included in this study. Each individual was sampled at five different skin sites at each time-point: the axillary vault (AV), the hypothenar palm (HP), the gluteal crease (GC), the nares (N) and the external auditory canal (EAC). The former three (AV, HP, GC) were subjected to the different bathing strategies on a daily base, while the other two sites (N, EAC) were not included in the bathing protocol. The sampling sites were selected based on their differential microbial communities, thus covering a wide spectrum of skin microbes [17]. The study was designed and performed in accordance with the Declaration of Helsinki and approved by the Ethics Committees of Charité– Universitätsmedizin Berlin (ID: EA1/1093/16).

### Sampling and DNA isolation

Sampling was conducted by trained personnel using swabs (Eurotubo Deltalab, Spain). Before obtaining samples from the skin, sterile swabs were moistened with PBS buffer. Subsequently, each swab was placed into a labeled 1.5 ml Eppendorf tube containing 300 µl of PBS and promptly frozen at −80°C for future analysis. The PBS tube utilized for moistening served as a blank control and underwent simultaneous processing alongside all other samples collected during the same sampling event.

DNA isolation was performed employing the ZymoBIOMICS DNA Miniprep Kit (Zymo Research, USA), with small adjustments to the manufacturer's lysis protocol aimed at enhancing DNA yield and facilitating comprehensive coverage of gram-positive bacterial species. Specifically, 250 µl of the sample was transferred to a ZR Bashing-Bead Lysis Tube, supplemented with 750 µl of Lysis Solution, and subjected to homogenization using a SpeedMill Plus (Analytik Jena, Germany) with a 5-min program operating at maximum speed. Subsequent binding and washing steps strictly followed the manufacturer's protocol. Elution was achieved through the addition of 50 µl of preheated (60 °C) DNase/RNase-free water, a process repeated twice to ensure thorough elution.

### Bacterial biomass measurements (qPCR and contact plates)

The bacterial biomass in the samples was measured 16S rRNA gene copies quantification using a qPCR approach as described elsewhere [18, 19]. In brief, 4 μl DNA input was subjected to a 20 μl SYBR-Green-based qPCR reactions (BioLine, UK) with 200 nM specific amplification primers for the V4 region of the 16S rRNA gene (515Fw: 5’-GTGYCAGCMGCCGCGGTAA-3’; 806Rv: 5’- GGACTACNVGGGTWTCTAAT-3’). The reactions were set up in a CAS-1200 pipetting robot (Qiagen, Netherlands) and run on a Rotor-Gene Q cycler (Qiagen, Netherlands). The cycling conditions included an initial denaturation step (95 °C, 10 min) and 40 amplification cycles (95 °C, 15 s; 58 °C, 20 s; 72 °C, 30 s). Non-template controls were included in each run to control for potential contamination. Quantification of absolute target copy numbers was performed using the standard curve method as implemented in the Rotor Gene Series software v. 2.1.0 (Qiagen, Netherlands). To validate the qPCR measurements, RODAC (Replicate Organism Detection and Counting) plates, provided by SPL Life Sciences, were utilized for monitoring bacterial surface colonization on two accessible skin sites (hand palm and axilla). The 5.5 cm diameter plates were applied directly to the skin surface for 5 s. After sampling, the plates were immediately covered and incubated at 37 °C for 18–24 h. Following incubation, colony-forming units (CFUs) were counted either immediately or within 48 h after storage at 2–8 °C. Growth attributed to a single spreader was recorded as one CFU.

### Library construction and 16S rRNA gene amplicon sequencing

16S rRNA amplicon library construction and sequencing were performed following the guidelines of Caporaso and Walters et al. [20, 21], as described in detail elsewhere [18, 19]. In summary, PCR amplification utilized R806- and F515-fusion primers with Golay-barcodes. The 50 μl PCR reaction was automated using a CAS-1200 pipetting robot (Qiagen, Netherlands) and conducted on a Thermal Cycler S1000 (BioRad, USA) using the Platinum PCR SuperMix (Thermo Fisher Scientific, USA). Thermal cycling comprised an initial denaturation step (94 °C, 3 min), followed by 35 amplification cycles (94 °C, 15 s; 58 °C, 20 s; 72 °C, 30 s), and a final elongation step at 72 °C for 10 min. PCR products were quantified using D1000 Tapes on a TapeStation 2200 (Agilent Technologies), pooled equimolarly, and subsequently purified via size-selection on 2% SizeSelect E-Gels (Thermo Fisher Scientific, USA). The resultant libraries were prepared for Illumina sequencing utilizing the MiSeq Reagent Kit v2 (Illumina, USA), and following the manufacturer’s guidelines. Sequencing reagents and the run plan were adjusted according to Caporaso et al. [20]. The library was supplemented with a 20% PhiX library (Illumina, USA) to account for low diversity issues during sequencing, and cluster density was maintained and 600–800 K/mm^2^. Sequencing was run on an Illumina MiSeq apparatus with 251 cycles.

### Sequencing data analysis

Raw reads underwent demultiplexing in QIIME 2 [22]. Following quality assessment, the q2-dada2 plugin was employed for denoising, chimeric sequence filtration, singleton removal, paired-end sequence merging, and dereplication of high-quality sequences [18]. Each sample yielded a minimum of 5000 reads, with an average sequencing depth of 91,551 reads per sample (post-quality filtering). More reads statistics are provided in Supplementary Table S2. Unless otherwise specified, default parameters were utilized for all DADA2 functions. Taxonomy assignment of the dada2-output feature table utilized a pre-trained Naive Bayes classifier trained on the SILVA REF NR 99 database (release 132), with sequences extracted using the 515F forward (GTGYCAGCMGCCGCGGTAA) and 806R reverse primer (GGACTACNVGGGTWTCTAAT) targeting the V4 region of the bacterial 16S rRNA gene [19]. Taxonomic classification employed the sub-classifying genus option within the SILVA database to retain data integrity and enable differentiation among bacterial strains within the same genus. Unspecified taxa resulting from sub-classification were assigned numerical identifiers. The datasets generated in this study are accessible at the SRA database under Bioproject accession number: PRJNA1111699 [https://www.ncbi.nlm.nih.gov/sra/PRJNA1111699]. Co-occurrence network analyses were performed to measure non-random interactions between bacteria at each skin site. These were generated with the SparCC software v.0.1.0 [23].

### Antibiotic resistance gene (ARG) detection

A total of 12 ARGs (see Suppl. Table S1) conferring resistance to beta-lactams, quinolones or polymyxins were screened for their presence in the samples throughout the course of the study. The determinants addressed in the study were the following: *blaKPC, blaNDM, blaOXA48, blaVIM, blaCMY, blaGES, blaSHV, blaTEM, blaCTX-M1, qnrB1, mcr1* and *mecA*. These include many of the most relevant ARGs isolated from nosocomial pathogens, as reported in European Antimicrobial Resistance Surveillance Network (EARS-Net) [24]. For their detection, custom multiplex Taq-Man assays were performed as described elsewhere [18, 19]. The real time qPCRs were carried out using the RNA UltraSense One-Step Quantitative RT-PCR System Kit (Thermo Fisher Scientific, USA) and analyzed using the Rotor-Gene 6000 software v.2.1.0 (Qiagen, Netherlands). DNA samples obtained from bacteria positive for the different ARGs were used as controls in each PCR. Samples were considered as positive for a particular ARG when the cycle threshold (Ct) was < 35.

### Statistics

Comparative analyses involving alpha-diversity metrics (Shannon Index) were performed using parametric approaches (ANOVA). For beta-diversity analyses, the evaluation of the dispersion between groups (*betadisper*) was followed by Permanova in order to detect significant clustering patterns between groups. These comparisons were performed pairwise (between two sites within each time-point). Dimension reduction for the potential detection of differentially distributed ASVs was achieved using ANCOM [25]. The significance threshold for all statistical tests was set at *p* < 0.05. Statistical analyses and graphic presentations were performed using GraphPad Prism (GraphPad Software, USA).

## Results

### Impact of the bathing strategies on the bacterial biomass

In the Chlorhexidine group, 7 patients were included to our analysis, in the Octenidine group 10 patients and in the control group 9 patients. Quantitative analysis of the samples taken from different skin sites at admission allowed us to confirm that the three intervention sites (AV, HP and GC) covered a broad spectrum of bacterial biomass found on the skin. The hypothenar palm displayed the lowest biomass, whereas the gluteal crease samples showed the highest 16S rRNA counts (Suppl. Fig. S2). The nares (N) and the external ear canal (EAC) showed a moderate range of bacterial load. These two sites are particularly interesting as they allow for the observation and longitudinal comparison of bacterial colonization patterns over time in the absence of any washing interventions, providing insights into the natural dynamics of the skin microbiome for these particular sites.

**Fig. 1:**
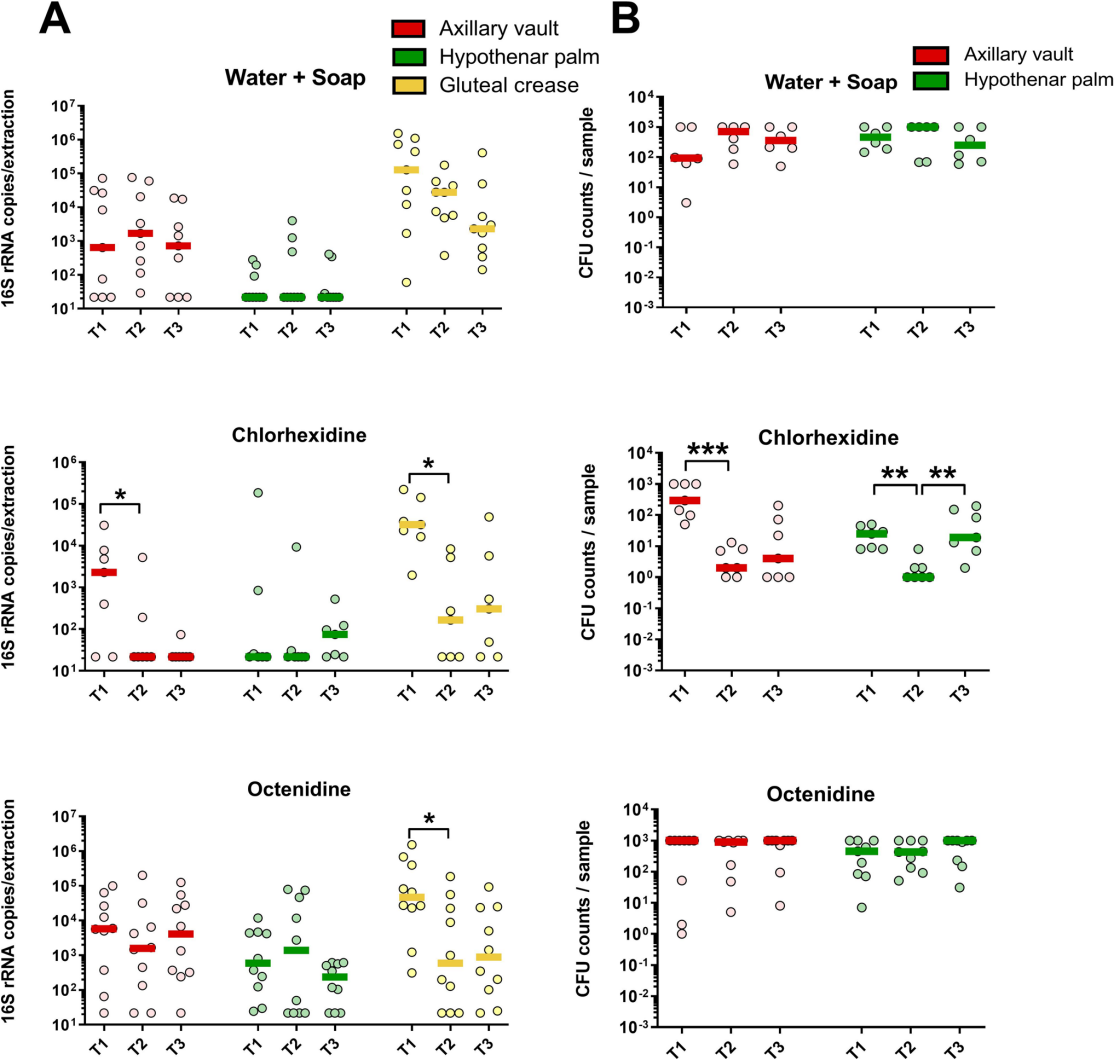
Quantitative analysis of the bacterial biomass on the skin after different bathing strategies. The scatter dot plots (with median) show the quantitative values obtained after 16S rRNA qPCR (**A**) and CFU counting (**B**), of the samples obtained from different skin sites at admission (T1), during ICU stay (T2) and after discharge (T3) (*p < 0.05; **p < 0.01; ***p < 0.001)

Further qPCR analyses of the 16S rRNA copies in all samples were then addressed to estimate the bacterial biomass present at the different skin sites throughout the intervention (across the three study time-points). The results showed that in contrast to the control strategy (water and soap bathing), the strategies involving disinfection (either with Chlorhexidine or Octenidine) did lead to a significant reduction of the bacterial biomass as measured by qPCR for specific sites. These reductions were particularly notable in the gluteal crease (GC) when analyzed by PCR and were maintained five days after discharge (T3). Chlorhexidine also showed reductions in the gluteal crease and the axillary vault (AV) at T2, with these reductions persisting at T3 (Fig. 1A). In contrast, washing with water and soap displayed a trend towards reduction in the gluteal crease, though this did not achieve statistical significance (Fig. 1A).

To validate these findings, the axillary vault (AV) and the hypothenar palm (HP) were sampled using RODAC plates and subjected to CFU counting (Fig. 1B). A significant reduction of CFUs could be confirmed at both tested sites at T2 after Chlorhexidine bathing (AV, *p* < 0.001; HP, *p* < 0.01; see Fig. 1B), but not for Octenidine or water & soap. In the case of the HP samples, the bacterial load at T3 returned to levels observed at admission (T1).

### Compositional analysis of the skin microbiome upon intervention

Analysis of the 16S rRNA sequencing data provided a detailed taxonomic overview of the bacterial communities on the skin of in total 26 ICU patients subjected to different bathing strategies in the ICUs of the three different hospitals. This analysis revealed high interindividual variance across all skin sites at the three time-points (T1, T2, T3) (see suppl. Fig. S3). However, Bray–Curtis beta-diversity analyses did not reveal any significant differences between different hospitals across the whole datasets (pairwise Permanova analyses, see suppl. Fig. S4). When specifically analyzing differential effects by the different bathing strategies on the main bacterial taxa found (relative abundance > 0.01), ANCOM analyses did not retrieve any significant changes in the taxonomic distribution after bathing (T1 vs. T2; T1 vs. T3) for any of the strategies and at any of the skin sites. Thus, we could not identify any specific taxonomic pattern change driven by any particular bathing strategy, nor any bacterial taxa consistently affected by any of the interventions.

**Fig. 2:**
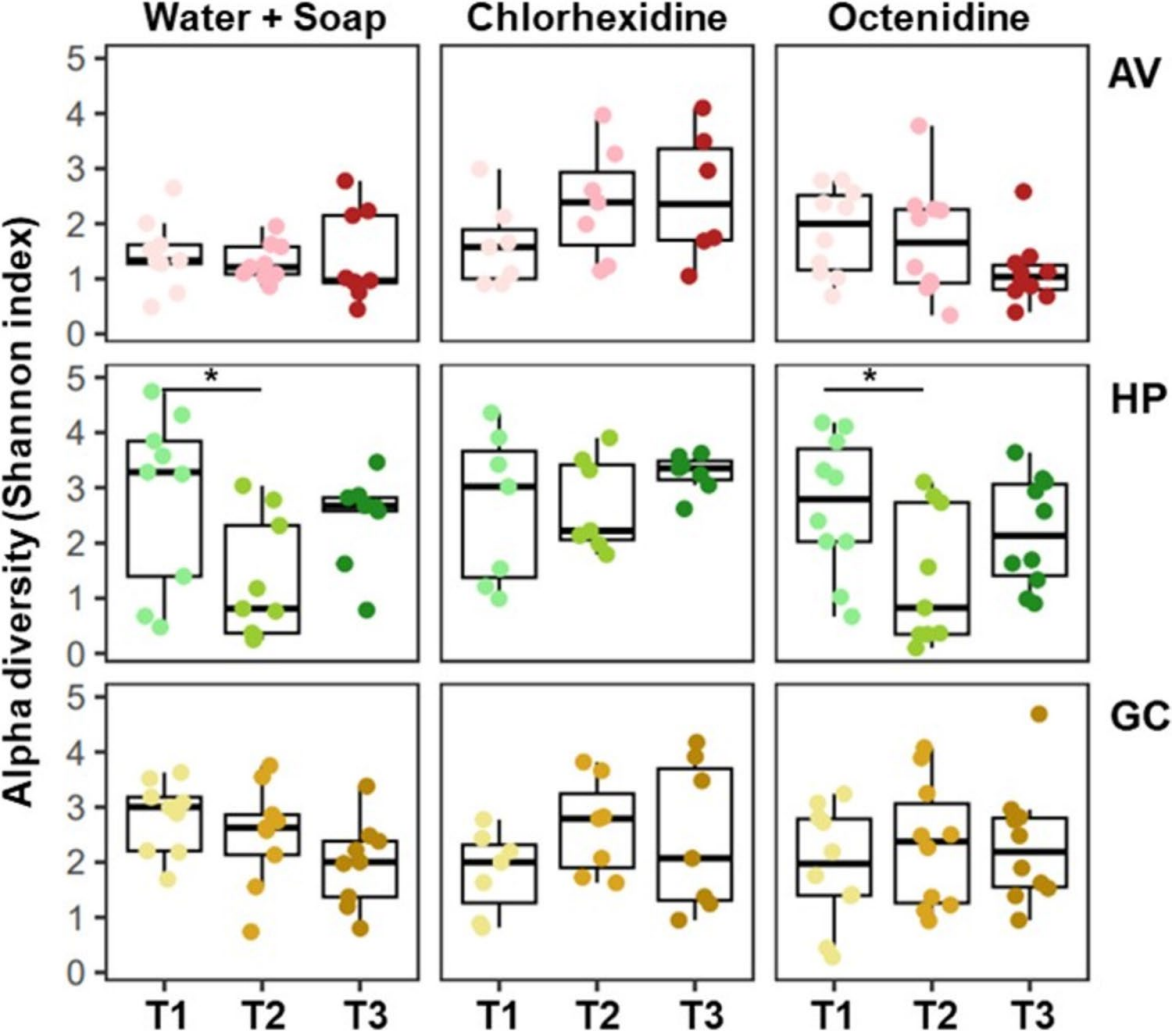
Alpha-diversity metrics of the skin microbiota upon different bathing strategies. Shown is the Shannon index (box and whiskers plot with median, ∗ *p* < 0.05, ANOVA) for each of the skin sites (axillary vault (AV), hypothenar palm (HP) and gluteal crease (GC)) at each time point (T1, T2, T3) of the intervention with the different bathing strategies

Analysis of the diversity metrics revealed a significant reduction in the alpha-diversity of the bacterial communities on the handpalms of patients that underwent bathing with either water and soap or Octenidine (T1 vs T2, *p* < 0.05). In contrast, Chlorhexidine-bathing did not lead to any changes in the alpha-diversity during ICU stay (T2) nor after discharge (T3) in the cohort subjected to this strategy (Fig. 2). These findings were supported by the results obtained by co-localization networks of the handpalm samples, where the complexity of the networks (nodes and edges) was conserved across the study for Chlorhexidine intervention while temporarily reduced during the ICU stay for patients with water and soap or Octenidine bathing (Suppl. Fig. S5). The skin sites not subjected to intervention (N and EAC) did not show any changes in their alpha-diversity (see Suppl. Fig. S6).

### Longitudinal impact of bathing interventions on skin microbiome site-specificity loss. 

**Fig. 3:**
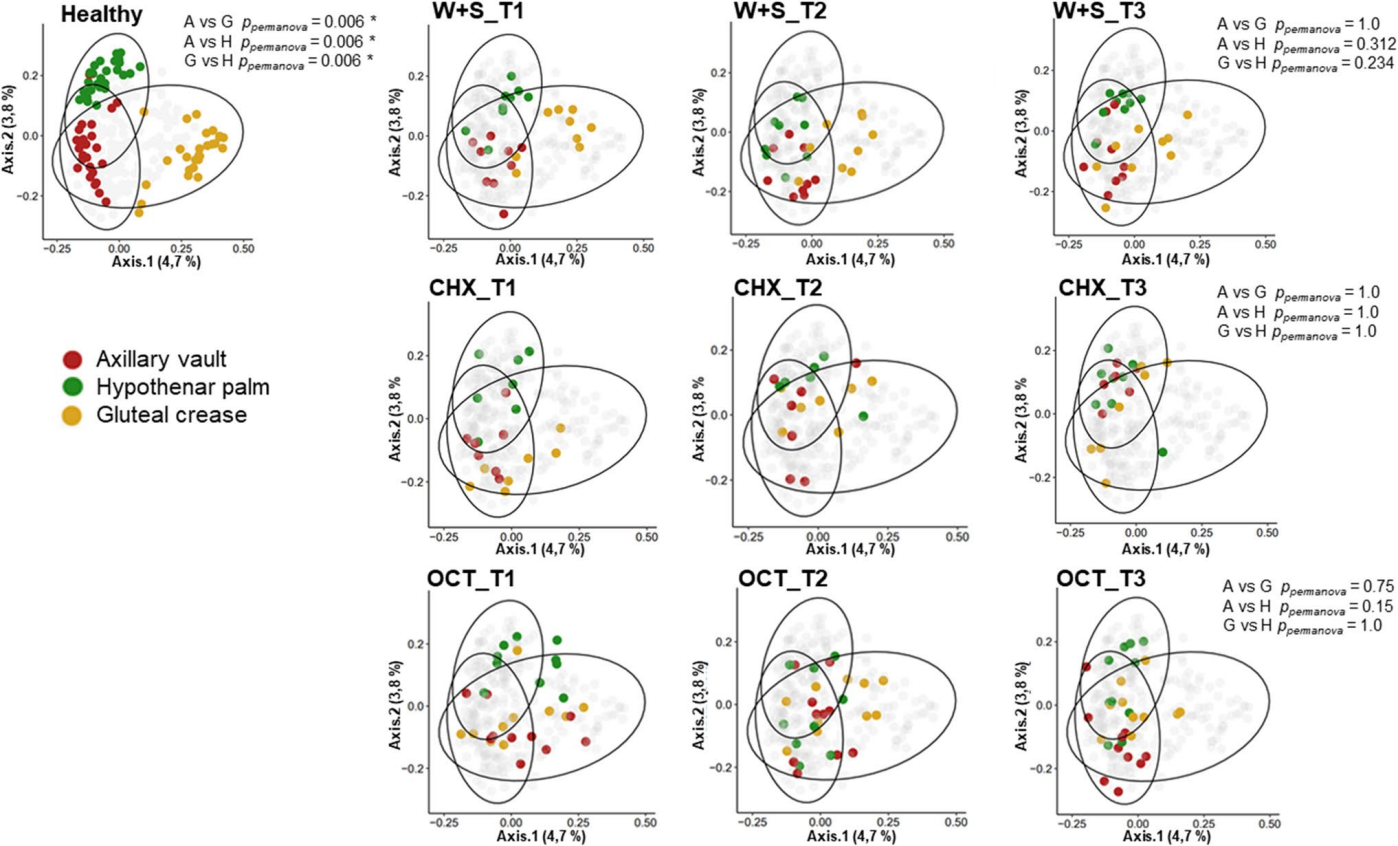
Site specificity of the bacterial communities on the skin as measured by beta-diversity metrics. Shown are the principal coordinate analyses of the β-diversity of the skin microbiome using Bray–Curtis distances for each of the skin sites in a healthy controls group (**A**) and in the ICU-patients cohort (**B**). The patients group samples were divided in the results obtained across the longitudinal study before (T1) and after intervention (T2: ICU stay; T3: after discharge). Pairwise comparisons between sites were made using Permanova tests. (WS: Water and soap; CHX: Chlorhexidine; OCT: Octenidine). Data for thse analysis of the healthy cohort was retrieved from Klassert et al. [5]

Next, we conducted comparisons between the three different time points for both washed and unwashed skin sites in order to observe the development of site-specific bacterial communities. Our previous analyses reported a loss of site specificity in ICU patients upon admission. Thus, we aimed to investigate whether this condition was reversed during ICU stay or after discharge upon the different bathing strategies. Beta-diversity revealed that the loss of site-specificity between different skin locations in ICU patients remained unaltered across all time points, regardless of whether sites were washed or which product has been used (Fig. 3; see also Suppl. Fig. S7 for non-washed sites). This uniformity across different skin sites indicates that the microbial communities on ICU patients' skin tend to lose their distinct, site-specific profiles that are reported in healthy individuals. This pattern was observed before the initiation of any bathing strategy and persisted irrespective of the bathing methods used throughout the ICU stay and beyond discharge. The consistent loss of site-specific microbial characteristics suggests a broader shift in the skin microbiome dynamics of ICU patients, likely driven by multiple factors beyond the specific bathing interventions.

### ARG dissemination in ICU patient skin microbiomes upon different bathing interventions. 

**Fig. 4:**
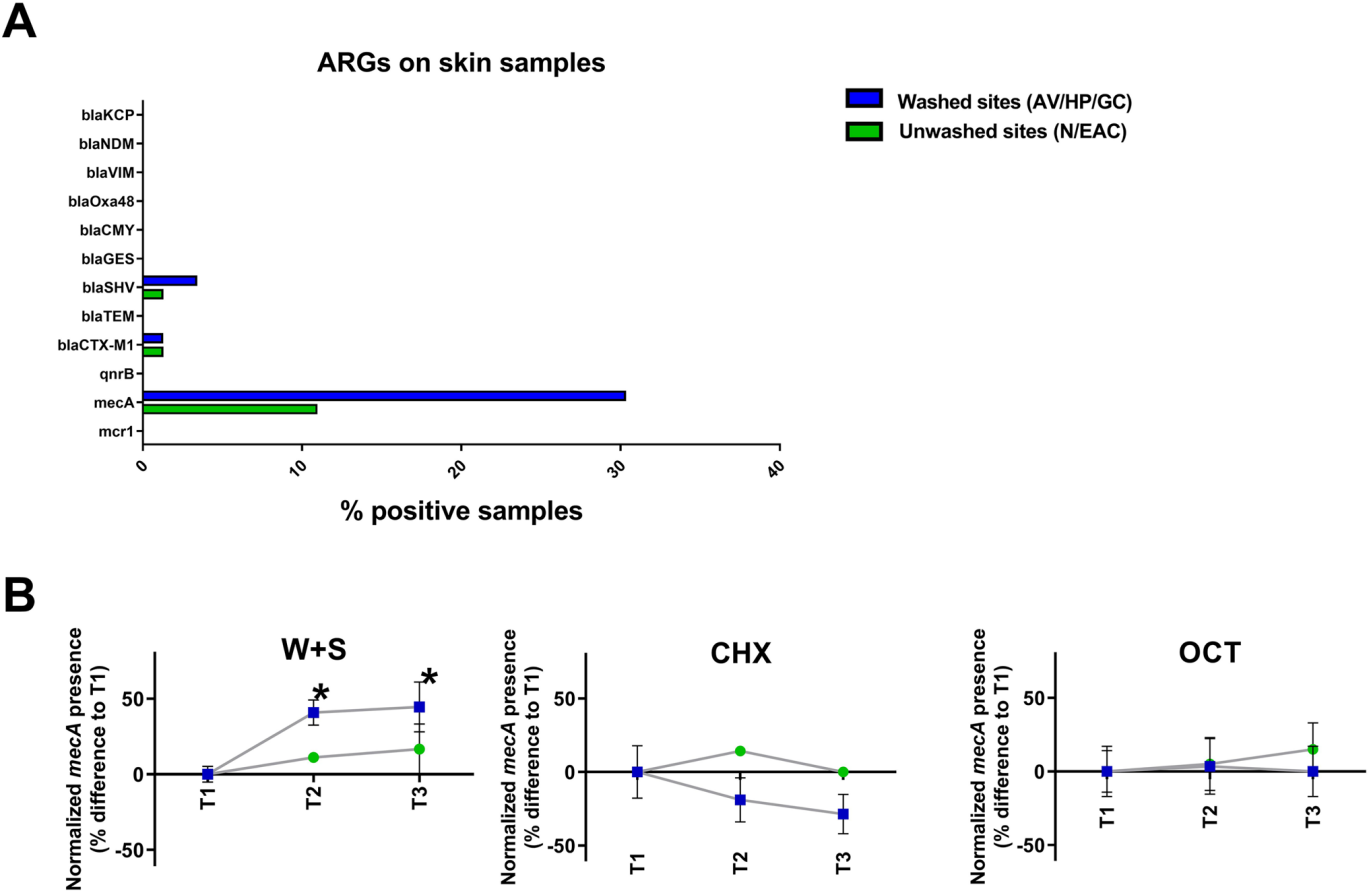
Antibiotic resistance gene (ARG) detection on the skin microbiome of ICU patients. **A** Bar chart depicting the ARG expression across all samples of our ICU cohort. Bars represent percentage of samples with positive ARG detection in sites that were subjected (blue) and not subjected (green) to bathing. **B** *mecA* detection across different bathing strategies. Shown is the normalized *mecA* presence (% change to T1) during ICU-stay (T2) and after discharge (T3). Statistical evaluation (2-tailed t-test) was performed between washed and unwashed sites in each case (*p < 0.05). (W+S: Water and soap; CHX: Chlorhexidine; OCT: Octenidine)

Screening of ARGs in the bacterial communities across all time-points and interventions revealed the presence of three genes in our sample set: two genes encoding for beta-lactamases (*blaSHV* and *blaCTX-M1*) and the gene encoding the low affinity penicillin-binding protein PBP2a (*mecA*), and which is part of the MGE cassette found in MRSA strains (Fig. 4A). Interestingly, especially *mecA* showed a much higher detection on sites that were washed (AV, HP or GC; 30.3% of positive samples) when compared to sites that did not undergo the intervention (N, EAC; 10.9% of positive samples). Thus, we investigated whether the presence of *mecA* was differentially impacted by the bathing strategies. As shown in Fig. 4B, the normalized *mecA* presence in washed sites was increased over its detection at admission (T1) when compared to unwashed skin sites (*p* < 0.05) at T2 and also T3 in the cohort subjected to water and soap bathing. In contrast, no significant changes were detected in washed sites across the interventions involving disinfection bathing (Chlorhexidine and Octenidine). The differential effect between bathing strategies was however not related to absolute changes in *Staphylococcus aureus* presence in the skin microbiome, as verified by specific *S. aureus gyrB* gene detection (Suppl. Fig. S8).

## Discussion

This longitudinal study characterizes the compositional changes of the skin microbiome of ICU patients upon different bathing strategies. Our results show that the loss of microbiome site-specificity observed on the skin of ICU patients remained unchanged independently from the bathing strategy applied. Indeed, the dysbiotic condition persisted even post-discharge, prompting significant inquiries into the restoration dynamics of microbiome homeostasis following ICU stay. In addition, Chlorhexidine bathing led to a sustained reduction of the bacterial biomass on different skin sites, while the microbial structures remained largely unaltered both in their diversity and their taxonomic composition. Further, antiseptic bathing with Chlorhexidine or Octenidine did not lead to an increased abundance of antiseptic resistance genes.

Maintaining the equilibrium of the skin microbiome is paramount for reducing infection risks, particularly among critically ill patients in ICUs. The skin, serving as the body's primary barrier against pathogens, relies on colonization resistance—a delicate interplay between commensal and pathogenic microorganisms—to prevent infections [6]. Disruptions to this microbial balance could compromise the skin's protective functions, rendering ICU patients more susceptible to nosocomial infections [15]. Thus, our investigation aimed at answering the question whether antiseptic intervention might lead to unintended changes in the bacterial community composition of the skin when compared to standard water and soap bathing.

The results of this study showed that antiseptic bathing was able to significantly reduce the bacterial biomass in the gluteal crease (for Chlorhexidine and Octenidine) and in the axilla (for Chlorhexidine only) as measured by qPCR of 16S rRNA copies. However, while reducing the number of gene copies during ICU-stay, none of the interventions led to an absolute decolonization. These results are in line with previous studies that show that transient reduction of bacterial biomass can be achieved by antiseptic bathing, but complete eradication of the skin microbiota is not achieved by topical decolonization approaches, neither in healthy nor hospitalized adults [3, 26–29]. One reason for this observation may rely on the resilience of the skin microbiota and their ability to recover from perturbations [29, 30]. Acosta et al. (2023) could recently demonstrate in a perturbation-recovery study, that the skin microbiome is extraordinarily stable even upon aggressive perturbation. The authors showed that viable skin-associated bacteria are predominantly located in cutaneous invaginations and hair follicles and that bacterial populations on the surface are rapidly replenished by these stable underlying communities [30]. Interestingly, their study also showed that DNA-based studies may thereby over-represent the viable skin microbiome [30]. Thus, in our study we aimed to validate the antiseptic-induced reduction observed at DNA level with culture-based methods. Bacterial growth on contact-plates confirmed the findings obtained for Chlorhexidine, but not Octenidine, in their ability to reduce bacterial biomass in our setting. These dissimilar results might be explained by the different concentrations used with both antiseptics (2% Chlorhexidine and 0.08% Octenidine) in this study. Although Octenidine demonstrates strong antiseptic efficacy at lower concentrations in vitro, the substantial difference in concentrations between the ready-to-use mitts may have implications in its effectiveness in clinical use [31]. Thus, the low Octenidine concentration used in this study might have allowed for a faster replenishment of viable bacteria from hair follicles towards the skin surface.

Analysis of the compositional data of the microbial communities revealed that the overall structures were preserved and only marginally disturbed upon any bathing strategy. In our sample set we could not identify any directed shifts induced by any of the two antiseptics (Chlorhexidine and Octenidine) which would have retrieved statistical changes in any particular taxa. While other studies have already reported body-site specific and taxa-specific shifts upon antiseptic agents on the skin [27], these are mainly observed in the very short term (within few hours after topical application). In our case, the sampling and microbiome analysis was performed 24 h after the previous bathing routine. Thus, our results line up with the findings from Mougeot et al. (2022), who observed only marginal changes in microbial communities on the skin from healthy volunteers 24–72 h after Chlorhexidine washing [32]. In addition, our data also showed that these microbial communities would remain relatively stable even 5 days after discharge of the patients. This raises the question on the time required by the skin microbiome of ICU patients to recover a healthy and balanced microbiome on different skin sites. As shown in our data, the dysbiosis and loss of site specificity remained unaltered across all time-points analyzed, independent of the bathing method used. More interestingly, for most sites the diversity metrics remained unaltered, and this was the case for both washed and unwashed sites. The only compositional factor that showed bathing-dependent changes was the alpha-diversity on the handpalms, the skin site with the lowest bacterial biomass. This might suggest, that on most skin sites, and especially those with higher biomass, bathing strategies do not have a major effect on microbiome dynamics, and that these might depend rather on host factors that could require a longer time period for the recovery of site-specific bacterial communities [6]. In addition, our study highlights that the skin microbiome of ICU patients may not have fully developed its protective functions, potentially contributing to increased susceptibility to infections or other complications observed in patients post-ICU [33].

When analyzing the dissemination of ARGs over three different time-points, a significant increase of the *mecA* determinant was observed in the cohort washed with water and soap. It is notorious, that unwashed skin sites (not subjected to any antiseptic cleaning method) in the same setting did not show the same increase in ARGs. It is however unclear whether the soap bathing might have potentially contributed to the increased presence of *mecA* in washed samples or if other factors might have contributed to this observation. On one side, we could not detect any significant increase of *S. aureus* in our specific qPCR approaches. On the other hand, we cannot exclude any potential local epidemiologic effects involving other *mecA*-carrying species nor potential selective effects by this bathing strategy without antiseptic supplementation. Importantly, the two antiseptic strategies (Chlorhexidine and Octenidine) did not show any significant increase or decrease in detected ARG over time. However, recent studies indicate that, in the long term, antiseptic washing may also have a potential for the selection of resistant strains and ARGs through mechanisms of cross-resistance with antibiotics. In recent years, this phenomenon has been reported with particular frequency in association with Chlorhexidine [12, 13, 34]. Thus, further comparative studies are very much needed and should include additional ARGs targeting all antibiotic groups for which cross-resistance has been reported.

This study has some limitations, such as the significant gap between the concentrations used for both antiseptic strategies. Further studies should include a wider range of concentrations for both products in order to improve the comparative analysis. Another limitation of this study is the restricted clinical data available for the ICU-patients, including any information about comorbidities or their history of antibiotic treatments. Thus, we were limited in the interpretation of interindividual differences in microbiome structures and were not able to link specific signatures with any clinical history or previous antibiotic treatment strategy. However, recent studies report negligible effects of systemic antibiotics on skin microbiome structures [35–37]. Another limitation is the center-specific intervention, which could introduce potential center effects, which may independently influence the microbiome. In addition, the relatively low sample size of this study may impact the effect sizes that can be addressed. Very small effects might remain unnoticed due to the high interindividual variation observed in the skin microbiome data. Further studies with additional clinical data, larger sample sizes, and more ARG targets should validate the findings made in this study. Data of supplementary MRSA screenings of the patients (e.g. nasal swabs) could thereby help to better understand individual and intervention-driven MRSA pattern changes. The main strength of this analysis is the longitudinal study design analyzing patient samples upon admission, during the ICU-stay and after ICU-discharge. However, and based on the data of this study, further investigations should include additional time-points after ICU-discharge, as it is not clear how long the skin microbiome of these patients might need for full recovery of site-specific community structures.

## Conclusion

Daily patient bathing with 2% Chlorhexidine impregnated cloths or 0.08% Octenidine wash mitts did not impact skin microbiome structures and antibiotic resistance gene accumulation in ICU patients when compared to non-antiseptic water and soap bathing routine. In fact, the loss of microbiome site-specificity observed on the skin of ICU patients remained unchanged independently from the bathing strategy applied. In addition, Chlorhexidine bathing led to a sustained reduction of the bacterial biomass on different skin sites, while the microbial structures remained largely unaltered both in their diversity and their taxonomic composition.

## Supplementary Information

Below is the link to the electronic supplementary material.

Supplementary file1 (PDF 1627 kb)

## Data Availability

The datasets generated in this study are accessible at the SRA database under Bioproject accession number: PRJNA1111699 [https://www.ncbi.nlm.nih.gov/sra/PRJNA1111699].
