# Supplementary material for: Short- and long-term impact of aseptic bathing strategies on the skin microbiome in ICU patients
Source: Med Microbiol Immunol. 2025 Jul 22;214(1):34. doi: 10.1007/s00430-025-00843-1 (PMC12283771; doi:10.1007/s00430-025-00843-1)
Supplement: Supplementary file 1 — Supplementary file1 (PDF 1627 kb) [file 430_2025_843_MOESM1_ESM.pdf]

SUPPLEMENTARY MATERIAL

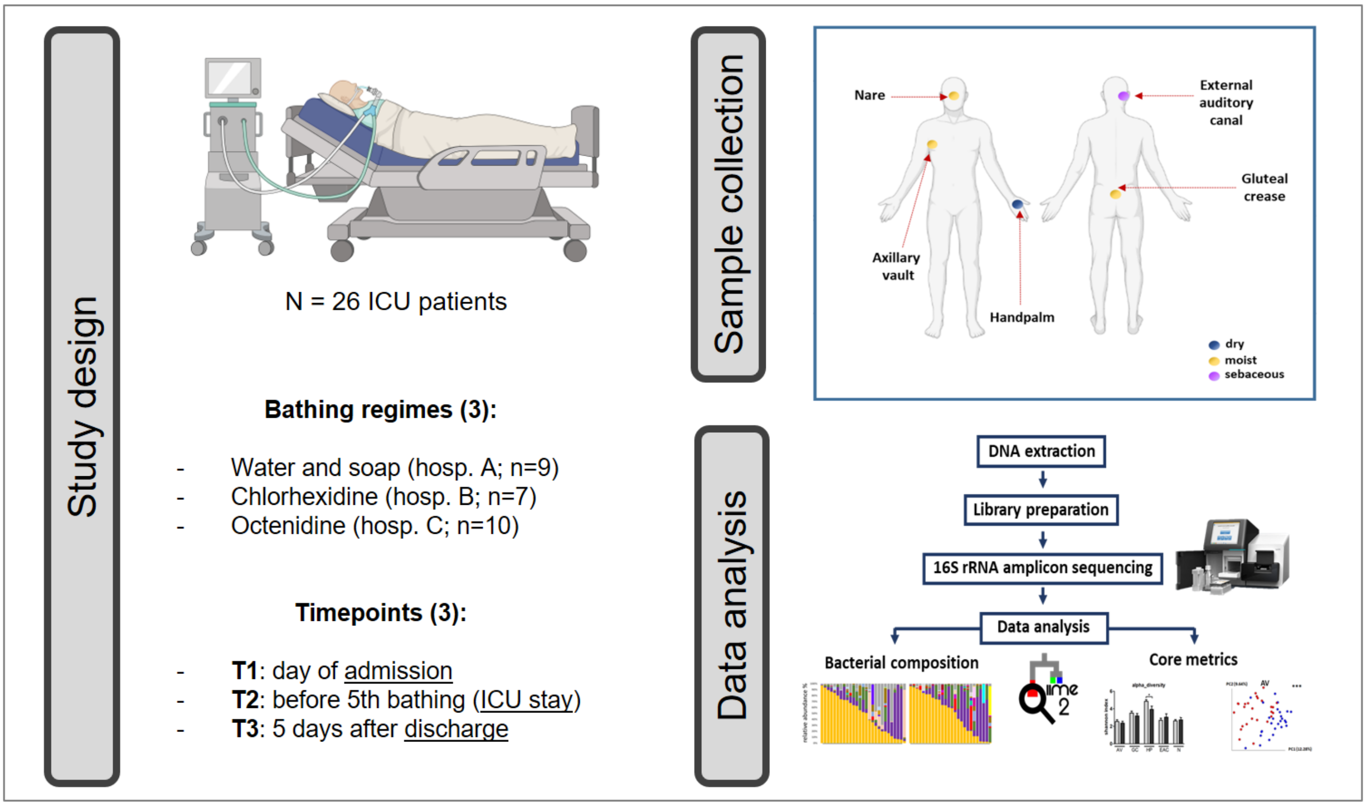

Suppl. Fig. S1.- Workflow schematic of the study. Overview of the study design (bathing regimes and time-points) as well as the samples collected and the data analysis. The three subcohorts were defined based on the bathing strategy in each of the hospitals: Water and Soap bathing in hospital A, Chlorhexidine bathing at the hospital B, and Octenidine bathing at the hospital C.

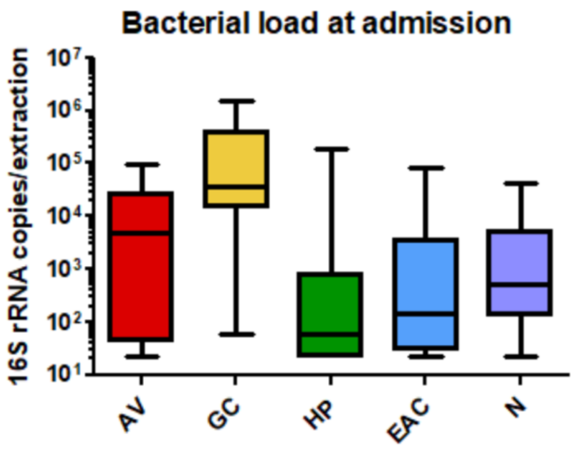

Suppl. Fig. S2.- Analysis of the bacterial load at the 5 tested skin sites of ICU patients at admission. Shown are the 16S rRNA copies (whisker box plot with median) as measured by qPCR for all five sites (AV: axillary vault, HP: hypothenar palm, GC: gluteal crease, EAC: external auditory canal, N: nares) for all 26 patients included in the study.

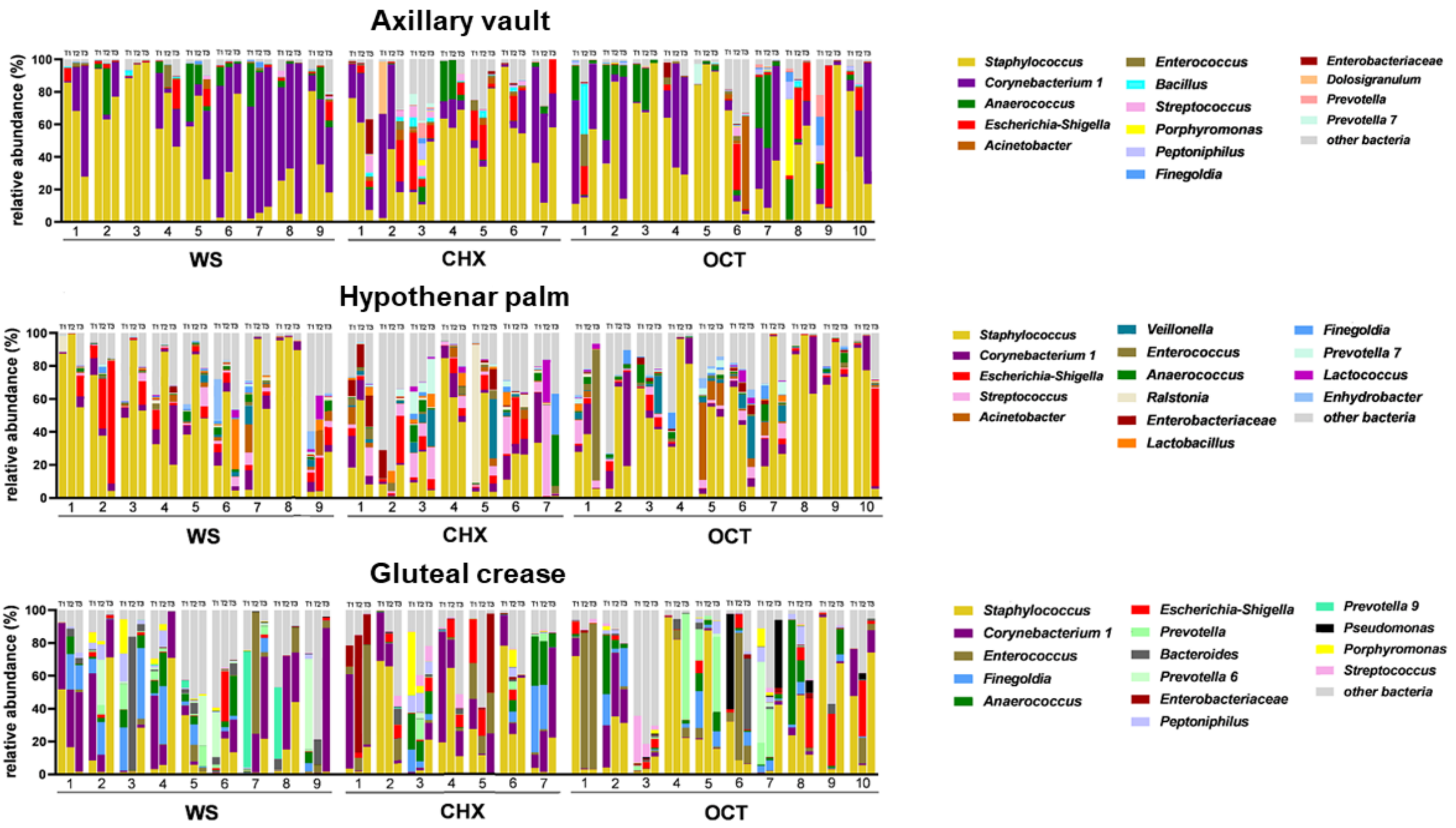

Suppl. Fig. S3.-. Colored bars depict the relative abundances of the 15 main taxa (>1 %) at genus level for each skin site. For each patient (numbers below bars) taxonomic data for all three time-points are shown (T1, T2, T3).

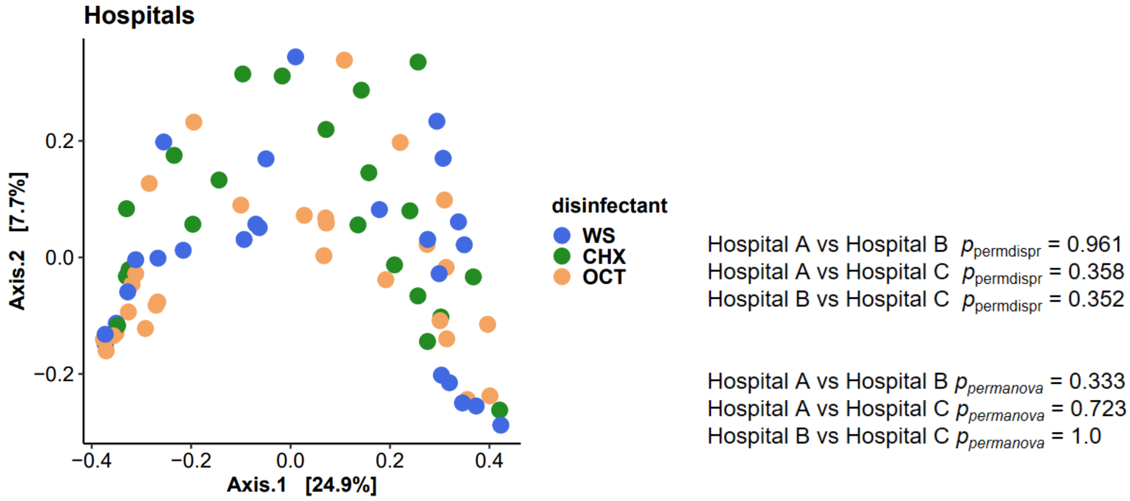

Suppl. Fig. S4.- Beta-diversity metrics of the skin microbiome from patients across the three hospitals (i.e. bathing strategies) addressed in this study. Shown is the principal coordinate analysis of the beta-diversity of the skin microbiome using Bray-Curtis distances. Comparative analyses (Permanova) were performed between the samples obtained from the three hospitals in which different strategies were investigated (WS/hosp A: water and soap; CHX /hosp B: Chlorhexidine; OCT/hosp C: Octenidine).

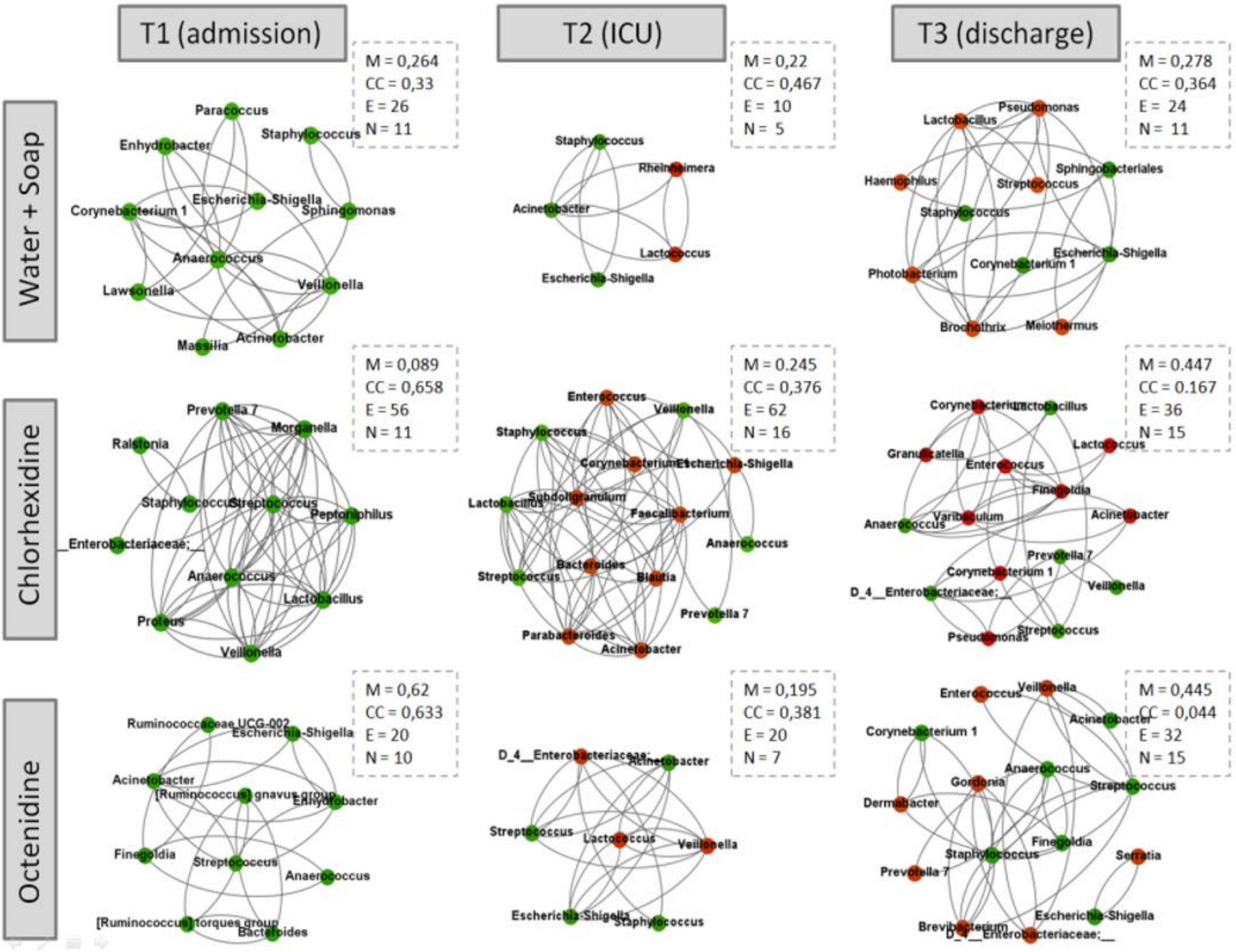

Suppl. Fig. S5.- Network analyses of the skin microbiome across different bathing strategies. Shown is the connectedness between nodes (colored according to their presence in the network at admission (green) or new integration (red)). The modularity index (M), the clustering coefficient (CC), the number of Edges (E) and the number of Nodes (N) are depicted for each of the networks.

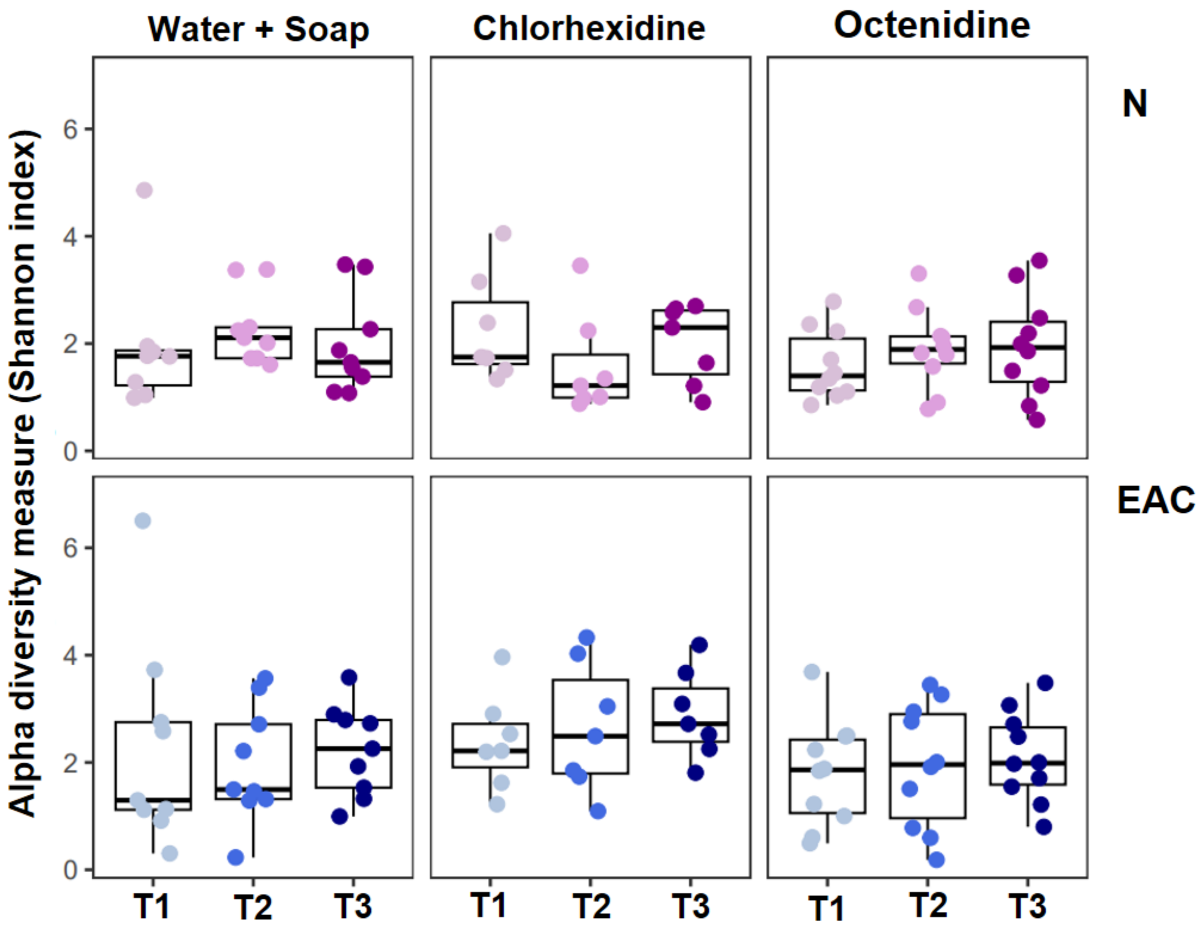

Suppl. Fig. S6.- Alpha-diversity metrics of the microbiota of unwashed skin sites. Shown is the Shannon index (box and whiskers plot with median) for each of the unwashed skin sites (nares (N), external auditory channel (EAC)) at each time point (T1, T2, T3) of the study. No significant differences were observed across the time-points for any of the sites or the cohorts (in which other skin sites were washed with either Water+Soap, Chlorhexidine, or Octenidine).

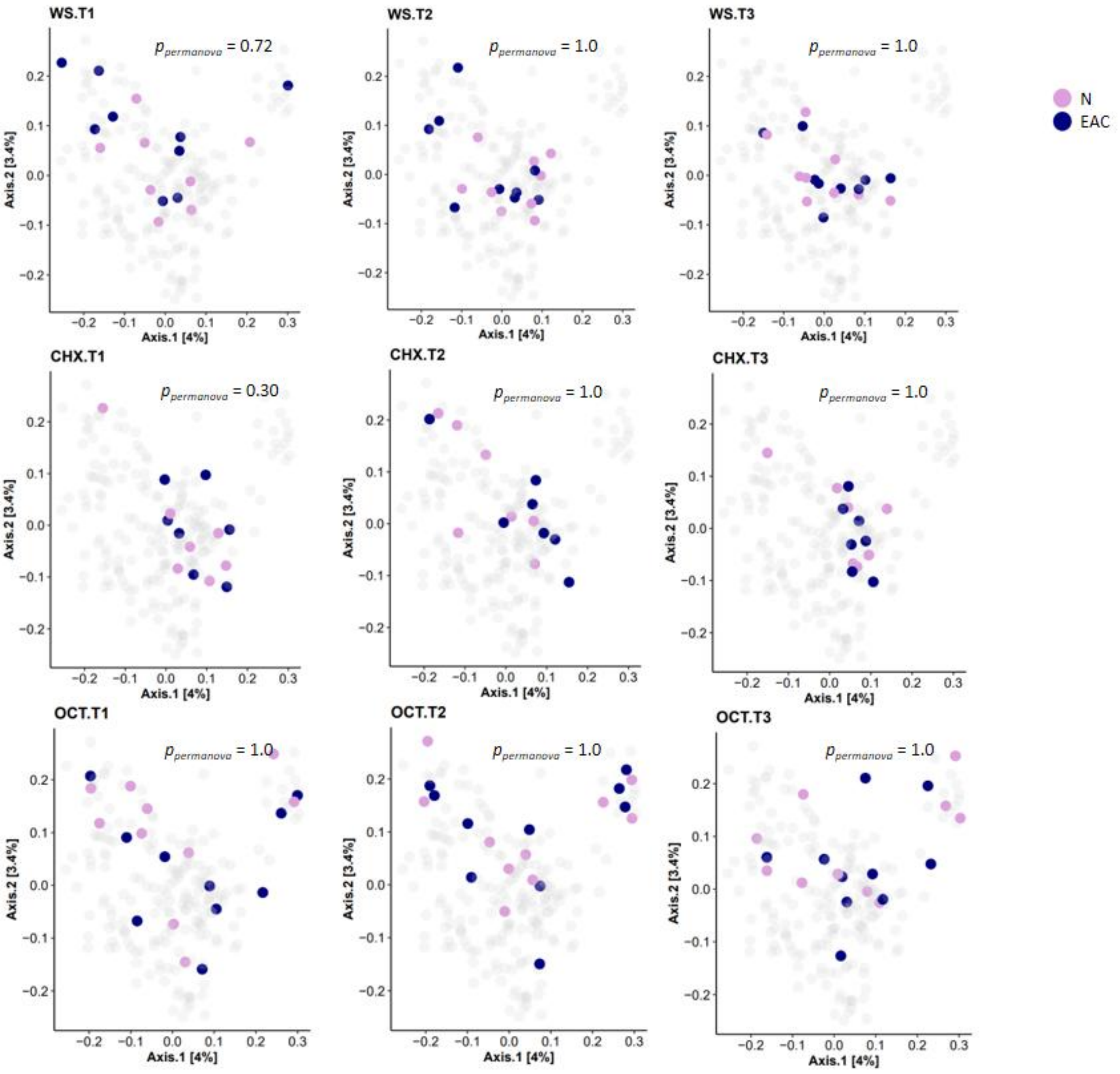

Suppl. Fig. S7.- Site specificity of the bacterial communities on the unwashed skin sites as measured by beta-diversity metrics. Shown are the principal coordinate analyses of the β-diversity of the skin microbiome using Bray-Curtis distances for each of the unwashed skin sites (N=nares, in violet; EAC=External Auditory Channel, in blue) in the ICU-patients cohort. The patients group samples were divided in the results obtained across the longitudinal study before (T1) and after intervention (T2: ICU stay; T3: after discharge). Pairwise comparisons between sites were made using Permanova tests. (The intervention groups were: WS=Water and soap; CHX=Chlorhexidine; OCT=Octenidine).

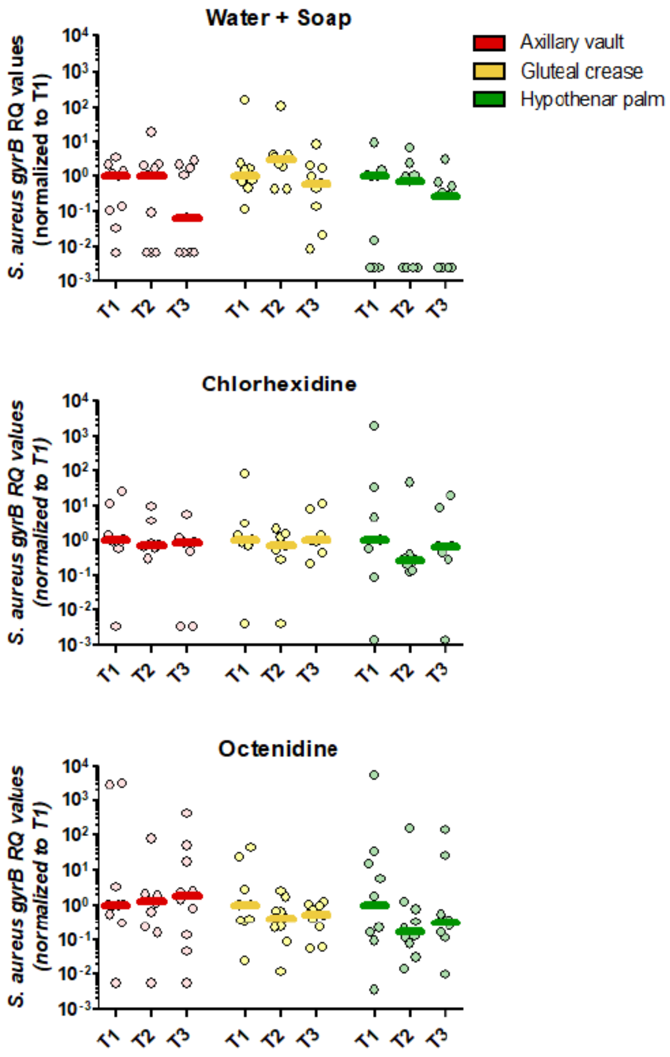

Suppl. Fig. S8.- Specific quantitative *S. aureus* detection in the skin microbiome samples. Normalized abundance (as fold change to the mean T1 values) of the *gyrB* gene copies detected by qPCR in each sample (dots). The line represents the mean value in each case.

Suppl. Table S1.- List of all ARGs addressed in this study, indicating the sequences of the designed primers and probes used for their detection via Taq-Man assay. In all cases, primers were designed to cover maximum number of known gene variants.

ARGs	Forward primers (5´-3´)	Reverse primers (5´-3´)	Reporters/Probes (5´-3´)	length	Dye
blaKPC	CTGTATCGCCGTCTAGTTCTG	AGTTTAGCGAATGGTTCCG	TGTCTTGTCTCTCATGGCCGCTGG	24	FAM
blaNDM	GCATTAGCCGCTGCATT	GATCGCCAAACCGTTGG	ACGATTGGCCAGCAAATGGAAACTGG	26	ROX
blaOXA48	TTCCCAATAGCTTGATCGC	CCATCCCACTTAAAGACTTGG	TCGATTTGGGCGTGGTTAAGGATGAAC	27	HEX
blaVIM	TGGCAACGTACGCATCACC	CGCAGCACCGGGATAGAA	TCTCTAGAAGGACTCTCATCGAGCGGG	27	Cya5
blaCMY	GGAGAAAACGCTCCAGCA	GGCCAGTTCAGCATCTCC	TCTCGCTACTGGCGTATTGGCGA	23	FAM
blaGES	GCCTATTGCTATGGCACGT	CTTTAGGAAAACCCGCTCGT	TGGCTGATCGGAAACCAAACGGG	23	ROX
blaSHV	CCGATGAACGCTTTCCCA	CGGCGAGTAGTCCACCA	CTGGCGCGGGTGGATGCC	18	Cya5
claTEM	GAGCTGAATGAAGCCATACCA	CCGCCTCCATCCAGTCT	GTTGCGCAAACTATTAACTGGCGAACTACT	30	FAM
blaCTX-M1	GGACGATGTCACTGGCTG	TCGTCTCCCAGCTGTCG	AGCGATAACGTGGCGATGAATAAGCTG	27	ROX
mcr1	GCGTTCAGCAGTCATTATGC	CGAGTAGATTGGCATGATCGG	GCATAAGCCGCTGCGTAGCTATGT	24	HEX
mecA	GATTCAGGTTACGGACAAGGT	GTGAGGTGCGTTAATATTGCC	ACCCAGTACAGATCCTTTCAATCTATAGCGC	31	HEX
qnrB	TCGACTTTCGACTGGCGA	AGCTTAACGCCTTGTAAATCAAC	CACACATTGCGATCTGACCAATTCGGA	27	Cya5

Suppl. Table S2.- Read statistics for the 457 samples (including blank controls) sequenced on an MiSeq apparatus in this study. Shown are the total and mean (per sample) input reads obtained for analysis and the reads recovered after quality filtering and merging paired-end reads.

Total input reads	48246768
Total merged reads (after dada2 pipeline)	41838616
Input (mean reads per sample)	105572,796
Mean merged reads per sample (after dada2 pipeline)	91550,5821
